# Supplementary material for: Chromothripsis during telomere crisis is independent of NHEJ, and consistent with a replicative origin
Source: Genome Res. 2019 May;29(5):737–49. doi: 10.1101/gr.240705.118 (PMC6499312; doi:10.1101/gr.240705.118)
Supplement: Supplemental Material [file supp_gr.240705.118_Supplemental_file_1.zip › contigs/annotated_contigs/DB50/contig.2.DB50_length_553_mean_cov_8.67992766727.docx]

**DB50_length_553_mean_cov_8.67992766727**

```
TTTGTCGCAAATGGAGTCTTCAGGAAGATATCCAGTCTGCAATAGCAAATGTGGAGGCCAGACTAAATGGAACGCCCCTGAGGAAATTA
 >chr16:17191517-17191808 + E=2e-163
TGGATGTAGCAGCAGTCCAGTTCACACGCTCATTCATTCATTCATCAAACACTGGCACAACATCTTACATTGTCAAGGCATGTAATGTG

TGCCGAGAATATAGACACTGTCCTTGCCTTCAAGGAACTTTCACCTTTGGAGTGAGAAGGCGAAAATAACTGATATTTACTGAGCTCTT

TCTGTATGCAGATACTAGGCTAT|C|AGTGTGGAGCCTGGAATACTGATGCTCCAGAAACGTGGAGAGAGATGCCGCTCTTCATGGCAG
                        >chr16:17192547-17192810 + E=1e-146
GTACGCAGGCCTTGCTTCCTGCAGCAAGTTAAAATCATCCACCATCATTAGCAAAGGGCTGTTACAAGGGCCTTTTTAAAGTGTCCTGT

GCCCAGGCTACAAGGAGCAATCCATCATGTTCCATAAGCATAAACACTCCACAGCTAATTAAGTGAGGGTGAAATCATCTGCACCATCC

ATGTGATATTTATTTAAAATG
```
